# Inactivation of Stat3 and crosstalk of miRNA155-5p and FOXO3a contribute to the induction of IGFBP1 expression by beta-elemene in human lung cancer

**DOI:** 10.1038/s12276-018-0146-6

**Published:** 2018-09-12

**Authors:** Fang Zheng, Qing Tang, Xiao-hua Zheng, JingJing Wu, HaiDing Huang, Haibo Zhang, Swei Sunny Hann

**Affiliations:** 1 Laboratory of Tumor Biology, Guangdong Provincial Hospital of Chinese Medicine, The Second Clinical Medical Collage, Guangzhou University of Chinese Medicine, 510120 Guangzhou, Guangdong Province China; 2 Department of Medical Oncology, Guangdong Provincial Hospital of Chinese Medicine, The Second Clinical Medical Collage, Guangzhou University of Chinese Medicine, 510120 Guangzhou, Guangdong Province China; 3 Laboratory Animal Research Center, Guangdong Provincial Hospital of Chinese Medicine, The Second Clinical Medical Collage, Guangzhou University of Chinese Medicine, 510120 Guangzhou, Guangdong Province China; 4 Guangdong Provincial Key Laboratory of Clinical Research on Traditional Chinese Medicine Syndrome, Guangdong Provincial Hospital of Chinese Medicine, The Second Clinical Medical Collage, Guangzhou University of Chinese Medicine, 510120 Guangzhou, Guangdong Province China

## Abstract

β-Elemene, an active component of natural plants, has been shown to exhibit anticancer properties. However, the detailed mechanism underlying these effects has yet to be determined. In this study, we show that β-elemene inhibits the growth of lung cancer cells. Mechanistically, we found that β-elemene decreased the phosphorylation of signal transducer and activator of transcription 3 (Stat3) and miRNA155-5p mRNA but induced the protein expression of human forkhead box class O (FOXO)3a; the latter two were abrogated in cells with overexpressed Stat3. Notably, miRNA155-5p mimics reduced FOXO3a luciferase reporter activity in the 3-UTR region and protein expression, whereas overexpressed FOXO3a countered the reduction of the miRNA155-5p levels by β-elemene. Moreover, β-elemene increased the mRNA and protein expression levels as well as promoter activity of insulin-like growth factor-binding protein 1 (IGFBP1); this finding was not observed in cells with a silenced FOXO3a gene and miRNA155-5p mimics. Finally, silencing of IGFBP1 blocked β-elemene-inhibited cell growth. Similar findings were observed in vivo. In summary, our results indicate that β-elemene increases IGFBP1 gene expression via inactivation of Stat3 followed by a reciprocal interaction between miRNA155-5p and FOXO3a. This effect leads to inhibition of human lung cancer cell growth. These findings reveal a novel molecular mechanism underlying the inhibitory effects of β-elemene on lung cancer cells.

## Lung cancer: How a herbal ingredient inhibits tumor growth

A compound found in one Chinese medicinal herb inhibits the growth of lung cancer cells by indirectly activating a protein with anti-proliferative properties. Hann and colleagues from the Guangzhou University of Chinese Medicine, China, uncovered the molecular pathways by which β-elemene, a natural compound isolated from the Curcuma wenyujin plant, mediates the anti-cancer effects. They showed that β-elemene inactivates the two important regulatory molecules, one protein and another small RNA, while also inducing the expression of one protein that promotes in killing cancer cells. These changes lead to elevated levels of the protein that prevents cell invasion and spread. Collectively, this altered signaling inside the lung cancer cell lead to reduced growth, in both cell-based culture and mouse model. The findings help explain why β-elemene has potential as a therapeutic agent in lung cancer.

## Introduction

Lung cancer is a prevalent malignancy and is considered a leading cause of cancer-related death in both males and females globally. Non-small cell lung cancer (NSCLC) represents ~85% of all lung cancer cases. In 2017, an estimated 222,500 new cases of lung cancer were diagnosed, and an estimated 155,870 deaths related to lung cancer were reported^1^. Substantial progress has been achieved in understanding the biological mechanism and comprehensive therapeutic options; despite this development, the 5-year survival rate remains low because of difficulty of early diagnosis, recurrence, metastasis, and overall ineffective therapies^1,2^. The current treatment paradigm for lung cancer shows a more challenging perspective and is greatly promoted by the discovery of oncogenic drivers and the development of targeted therapies directed specifically against these drivers. Nevertheless, the development of other therapeutic modalities for intervention against this malignancy is crucial.

β-Elemene is a major bioactive sesquiterpenoid isolated from the essential oils of *Curcuma wenyujin*, a Chinese medicinal herb against several cancer types. β-Elemene has been shown to inhibit various cancer types by regulating multiple signaling pathways and targeting genes or/and proteins without severe adverse responses^3–5^. β-Elemene upregulated endoplasmic reticulum stress-related proteins (ERs), such as protein kinase R-like endoplasmic reticulum kinase (PERK), inositol-requiring protein 1α (IRE1α), and activating transcription factor 6 (ATF6); meanwhile, β-elemene downregulated Bcl-2 expression, thereby inducing apoptosis in NSCLC cells. This finding suggested that ERs were activated via the PERK/IRE1α/ATF6-associated pathway^6^. We previously observed that β-elemene inhibited NSCLC cell growth via the extracellular signaling-regulated kinase 1/2 (ERK1/2)- and AMP-activated protein kinase α (AMPKα)-mediated inhibition of transcription factor Sp1 followed by a reduction in DNA methyltransferase 1 (DNMT1) expression^7^. Regardless, the detailed mechanisms underlying the effect and potential benefits of β-elemene, together with the occurrence, prevention, and treatment of NSCLC, remain undetermined.

Signal transducer and activator of transcription 3 (Stat3) belongs to the Stat family of transcription factors and is an important signaling molecule for many cytokines and growth factor receptors. Stat3 is constitutively activated in many human cancers involved in multiple biological functions, such as inflammation, proliferation, oncogenesis, anti-apoptosis progression, and metastasis in cancer^8,9^. Stat3 blockade has been shown to inhibit cell proliferation, induce apoptosis, and suppress tumor formation of cancers including lung cancer^10,11^. Thus, targeting this signaling pathway can be a potential therapeutic approach for the prevention and treatment of cancer^11–13^.

Human forkhead box class O (FoxO) transcription factors are activated in response to a wide range of stimuli, such as growth factors, insulin, nutrient levels, and oxidative stress. FoxO transcription factors play an important role in inhibiting tumor growth mainly by regulating genes involved in cell cycle arrest, as well as apoptosis and proliferation, among others^14,15^. Among the members of the FoxO class (FOXO1, FOXO3a, FOXO4, and FOXO6), FOXO3a is the most extensively studied member and is considered a tumor suppressor. Numerous studies have demonstrated that FOXO3a regulates a wide range of biological functions, including growth, differentiation, apoptosis, protection against oxidative stress, and metabolism^16–18^. Exogenous expression of FOXO3a inhibited tumor growth in several cancer types^19,20^. These studies suggested that FOXO3a could be an attractive therapeutic target for cancer treatment. However, FOXO3a was also found to promote cancer cell growth under oxidative stress^21^ and serum-deprived conditions^22^. Therefore, the true role of FOXO3a in cancer prevention and therapeutics could be more complex than thought and remains undetermined.

MicroRNAs (miRNAs) are small non-coding RNAs that regulate fundamental cellular processes at the transcriptional and translational levels. Dysregulation of miRNAs, which act as oncogenes or tumor suppressor genes, was found to be involved in the initiation and progression of several human cancers^23^. Among these miRNAs, miR155-5p was reported to be involved in several biological functions and regarded as a potential serum biomarker in patients with various types of cancer, including lung cancer^24,25^. In addition, miR155-5p promotes proliferation, invasion, and migration and inhibits apoptosis by targeting genes and regulating signaling pathways in different types of cancer^26,27^. This finding emphasizes the importance of miR155-5p in the occurrence and progression of cancer, suggesting that miRNA modulation could be a novel target for miRNA-based therapies for cancer. Regardless, the detailed molecular mechanisms underlying the regulation of lung cancer function have yet to be clarified.

Insulin-like growth factor-binding proteins (IGFBP)1 to IGFBP6 are high-affinity regulators of insulin-like growth factor (IGF) activity and modulate important biological processes, including cell proliferation, survival, migration, senescence, autophagy, angiogenesis, differentiation, and apoptosis^28–30^. IGFBP1 is abundantly expressed in the liver and decidualized endometrium^31^. Apart from inhibiting IGF actions by preventing binding to the IGF-I receptor, IGFBP1 also performs IGF-independent actions, such as the modulation of other growth factors, nuclear localization, transcriptional regulation, and binding to non-IGF molecules involved in tumorigenesis, growth, progression, and metastasis^30,32–34^. High IGFBP mRNA expression was highly correlated with good prognosis in a breast cancer mouse model^35^. We previously observed that ursolic acid, a natural pentacyclic triterpenoid, and emodin, a natural anthraquinone derivative isolated from the roots of *Rheum palmatum*, inhibited the growth of hepatocellular carcinoma and lung cancer cells by inducing IGFBP1 expression^36,37^. This finding suggested the tumor suppressor role of this molecule. However, conflicting results were found in other types of cancer such as prostate^38,39^, endometrial^40^, and others^41^. Thus, IGFBP1 can potentially exert dual effects on cancer cell motility and growth depending on the environmental content and cells tested^34^. Notably, the expression and function of IGFBP1 in stimulating or inhibiting lung cancer growth, as well as the detailed mechanism underlying the effect of β-elemene have yet to be elucidated.

In the current study, we further explored the potential mechanism by which β-elemene inhibits the growth of NSCLC cells. We proved that β-elemene increased IGFBP1 gene expression via inactivation of Stat3 followed by reciprocal interaction between FOXO3a and miRNA155-5p in vitro and in vivo.

## Materials and methods

### Reagents and cell culture

Monoclonal antibodies specific for total Stat3 and phosphor-Stat3 (Tyr705), as well as FOXO3a, were purchased from Cell Signaling Technology, Inc. (Beverly, MA, USA). IGFBP1 and GAPDH antibodies were obtained from Abcam (Cambridge, MA, USA). 3-(4,5-Dimethylthiazol-2-yl)-2,5-diphenyltetrazolium bromide (MTT) powder was purchased from Sigma-Aldrich (St. Louis, MO, USA). 5-Ethynyl-2′-deoxyuridine (EdU) detection kit and miR155-5p mimics and inhibitors were obtained from Ribo Biological Co., Ltd. (Guangzhou, China). FOXO3a and IGFBP1 small interfering RNAs (siRNAs) and Lipofectamine 3000 reagent were obtained from Life Technologies (Carlsbad, CA, USA). PCMV6-AC-GFP (control vector) and Stat3 overexpression plasmid were obtained from OriGene Technologies (Rockville, MD, USA). β-Elemene, which was purchased from Dalian Holley Jingang Pharmaceutical Company (Dalian, Liaoning, China), was freshly diluted to a final concentration with culture medium prior to experiments. A549 and H1975 human lung cancer cells were obtained from The Cell Bank of Type Culture Collection of Chinese Academy of Sciences (Shanghai, China) and authenticated for the absence of mycoplasma, genotypes, drug response, and morphology by using a commercial kit provided by Guangzhou Cellcook Biotech Co. Ltd (Guangzhou, China). Cells were cultured at 37 °C in a humidified atmosphere containing 5% CO_2_. The culture medium consisted of RPMI 1640 medium from Life Technologies (GIBCO, Grand Island, NY, USA) supplemented with 10% (v/v) fetal bovine serum (GIBCO, Grand Island, NY, USA), 100 µg/mL of streptomycin, and 100 U/mL penicillin. In addition, the A549-Luc medium was added with Geneticin G-418 Sulfate (Life Technologies, Carlsbad, CA, USA) at a concentration of 200 μg/mL. After reaching 80–90% confluence, the cells were digested with 0.25% trypsin for subsequent experiments.

### Cell viability assay

H1975 cells were seeded into a 96-well microtiter plate of 5 × 10^3^ cells/well and then treated with increasing concentrations of β-elemene for up to 72 h. Cell viability was determined by MTT assay following the method reported in a previous study^42^. Lastly, absorbance was measured at 570 nm using an ELISA reader (Perkin Elmer, Victor X5, Waltham, MA, USA). Each experiment was repeated three times. Cell viability (%) was calculated as follows: (absorbance of test sample/absorbance of control)  × 100.

### EdU incorporation assay

These kits measure cell proliferation by detecting the incorporation of the alkyne-modified nucleoside EdU into DNA by copper-catalyzed azide–alkyne click chemistry to attach fluorescent probes. A549 and H1975 cells were seeded in 96-well plates and treated with β-elemene (25 μg/mL) for 48 h. After the medium was discarded, 50 µM EdU was added for 2 h at 37 °C, fixed in 4% PBS, and stained with Apollo reaction reagent. All DNA contents of the cells were stained with Hoechst 33342. Pictures were taken at ×100 magnification using an inverted fluorescence microscope (Nikon, Ts2R-FL, Tokyo, Japan). At least three captured fields were randomly selected, and the EdU-positive cells were calculated as follows: percentage of EdU-positive cells = (EdU-positive cells/Hoechst stain cells) × 100.

### Quantitative real-time PCR

Quantitative real-time PCR (qRT-PCR) assay was conducted to examine miRNA155-5p expression, as well as the FOXO3a and IGFBP1 transcripts. Special primers of miRNA155-5p and U6 (endogenous gene) were purchased from GenePharma (Shanghai, China). These primers, together with the other primers used in this study, were designed as follows: miR155-5p forward 5′-GCTTCGGTTAATGCTAATCGTG-3′; miR155-5p reverse 5′-CAGAGCAGGGTCCGAGGTA-3′; U6-forward 5′-ATTGGAACGATACAGAGAAGATT-3′; U6-reverse 5′-GGAACGCTTCACGAATTTG-3′ FOXO3a forward 5′-GCAAGCACAGAGTTGGATGA-3′; reverse 5′-CAGGTCGTCCATGAGGTTTT-3′; IGFBP1 forward 5′-TCACAGCAGACAGTGTGAGAC-3′; reverse 5′-CCCAGGGATCCTCTTCCCAT-3′; and GAPDH (endogenous gene) forward 5′-AAGCCTGCCGGTGACTAAC-3′; reverse 5′-GCGCCCAATACGACCAAATC-3′. First-strand cDNA was synthesized from total RNA (1 μg) by reverse transcription using PrimeScript™RT Reagent Kit (Takara Bio, Inc., Kusatsu, Shiga, Japan) in accordance with the instructions provided by the manufacturer. Quantitative real-time PCR was performed in a 20 μL mixture containing 2 μL of the cDNA prepared using a SYBR®Premix Ex Taq™II Kit (Takara Bio, Inc., Kusatsu, Shiga, Japan) on an ABI 7500 Real-Time PCR System (Applied Biosystems, Grand Island, NY, USA). The PCR conditions were as follows: 30 s at 95 °C followed by 40 cycles for 5 s at 95 °C and 34 s at 60 °C. Each sample was tested in triplicate, and the results of each sample were normalized by an endogenous gene.

### Western blot analysis

After treatment with β-elemene, the cells were lysed with 1 × RIPA buffer. The protein concentrations were measured using the BCA Protein Assay Kit from Thermo Fisher Scientific. Equal amounts of protein from whole cell lysates were solubilized in 3 × SDS sample buffer and separated on 10% SDS polyacrylamide gels. Polyvinylidene difluoride (PVDF) membranes (Millipore, Burlington, MA, USA) were incubated with antibodies against phosphor-Stat3, total Stat3, FOXO3a, IGFBP1, and GAPDH at 4 °C overnight. Subsequently, the membranes were washed and incubated with a secondary antibody. The membranes were washed again and transferred to a freshly prepared enhanced chemiluminescence solution (Millipore, Burlington, MA, USA). Signals were then observed using the ChemiDoc XRS + System (Bio-Rad, Hercules, CA, USA).

### Transient transfection assays

A549 and H1975 cells were seeded at a density of 2 × 10^5^ cells/well in 6-well plates and grown to 50–60% confluence. The miRNA155-5p mimics or inhibitors and the negative controls were mixed with the ribo FECT™ CP transfection reagent (RiboBio Co., Guangzhou, China) following the instructions provided by the manufacturer; the cells were incubated with these mixed reagents for 48 h at 37 °C. In separate experiments, the desired pCMV6-AC-GFP or Stat3-GFP and IGFBP1-GFP overexpression plasmids purchased from OriGene Technologies, Inc. (Rockville, MD, USA), were transfected into the cells with Lipofectamine 3000 reagent at a final concentration of 1 μg/mL. The cells were incubated for 24 h at 37 °C followed by treatment with β-elemene for the indicated time for all other experiments.

### Luciferase reporter assay

Wild-type and mutated FOXO3a 3′-UTR luciferase vectors obtained from GeneCopoeia, Inc. (Rockville, MD, USA), were transfected with either miR155 mimic or negative control into the cells using Lipofectamine 3000 transfection reagent. The preparation of cell extracts and measurement of luciferase activities were determined using the Secrete-Pair™ Dual Luminescence Assay Kit (GeneCopoeia, Rockville, MD, USA). Luciferase activity was normalized with secreted embryonic alkaline phosphatase (SEAP) activity within each sample. In separate experiments, control and pGL3-IGFBP1 promoter plasmids purchased from GenePharma (Shanghai, China) were transfected with 0.1 µg of the Renilla luciferase reporter plasmid into the cells with Lipofectamine 3000. After 24 h, the cells were collected and lysed. Luciferase activity assay was conducted using the Dual-Luciferase Reporter Assay System (Promega, Beijing, China) in accordance with the instructions provided by the manufacturer.

### Transfection with FOXO3a and IGFBP1 siRNAs

FOXO3a, IGFBP1, and control siRNAs were purchased from Life Technologies (Carlsbad, CA, USA). For the transfection procedure, cells were seeded in 6- or 96-well culture plates, grown to 40–50% confluence, and then transfected using Lipofectamine 3000. Lipofectamine 3000 and siRNAs (up to 25 nM) diluted with Opti-MEM (Invitrogen, Carlsbad, CA, USA) were mixed and incubated for 15 min at room temperature and then added to the cells. The medium was replaced and cultured with or without β-elemene for all other experiments.

### Xenograft tumor study

All experimental procedures related to animals were performed in accordance with the guidelines for the care and use of laboratory animals approved by the Animal Care and Use Committee of Guangdong Provincial Hospital of Chinese Medicine (with Ethics Approval Number 2017036) and the National Institutes of Health Guide for the Care and Use of Laboratory Animals (NIH Publication No. 8023, revised 1978). A total of 20 female nude mice (weight of 18–20 g) were purchased from Beijing Vital River Laboratory Animal Technology Co., Ltd. (Beijing, China), and kept in a pathogen-free environment at the Animal Center of Guangdong Provincial Hospital of Chinese Medicine. A549 cells (2 × 10^6^) carrying the luciferase reporter gene (A549-Luc, Guangzhou Land Technology Co., Guangzhou, China) were cultured in medium with Geneticin (G-418, Sulfate, Life Technologies, USA) at a concentration of 200 μg/mL, resuspended in 0.2 mL of phenol red-free RIPM 1640 with 2% FBS, and subcutaneously injected into the flank region of the nude mice. Xenografts were expected to grow for seven days, when the initial measurements were available. Mice were randomly divided into the control and β-elemene (75 mg/kg) groups, which received treatment once a day via intraperitoneal injection for up to 16 days (*n* = 10/group). Mice were then anesthetized by inhalation of 2% isoflurane. The substrate D-Luciferin (Caliper Life Sciences, Hopkinton, MA, USA) was injected into the peritoneal cavity of the mice at a dose of 150 mg/kg in ~100 μL. The bioluminescence imaging signal was determined using the IVIS200 Imaging System (Xenogen/Caliper, Alameda, CA, USA). The formula for an oblong sphere: volume = (width^2^ × length) × 2 was used to measure the tumor volume. The body weights of the mice were measured once a week. All mice were sacrificed on day 16 in accordance with the Guide for the Care and Use of Laboratory Animals.

### Immunohistochemistry (IHC)

Immunohistochemical assays were conducted to determine the IGFBP1 protein expression in xenografted tumors (control and β-elemene-treated groups). Serial (4 mm) sections from paraffin-embedded conventional tissues were deparaffinized in xylene and hydrated in a series of graded alcohols. The antigen was retrieved using a citric acid buffer (pH 6.0) and immersed in 3% H_2_O_2_ to inhibit endogenous peroxidase activity followed by incubation in 5% bovine serum albumin to block nonspecific binding. Overnight incubation at 4 °C with a primary antibody against IGFBP1 (dilutions of 1:100, Abcam, Cambridge, UK) was performed followed by incubation with a secondary antibody (Maixin Biotech. Co., Ltd, Fuzhou, China) for 30 min. Detection was conducted using 3,3′-diaminobenzidine in accordance with the instructions provided by the manufacturer (DAB Kit, Maixin Biotech. Co., Ltd., Fuzhou, China). Pictures were taken with a microscope (×200 magnification, Olympus Corporation BX53 + DP72, Tokyo, Japan).

### Statistical analysis

Each experiment was repeated at least three times in triplicate. Continuous variables are presented as the mean ± SEM or mean ± SD from three independent experiments. Differences between groups were analyzed by one-way ANOVA, and post hoc Bonferroni analysis was conducted for multiple comparisons using GraphPad Prism 5.0 (GraphPad Software, La Jolla, CA, USA). *P* values < 0.05 were considered statistically significant.

## Results

### β-Elemene inhibited cell growth in lung cancer cells

**Fig. 1:**
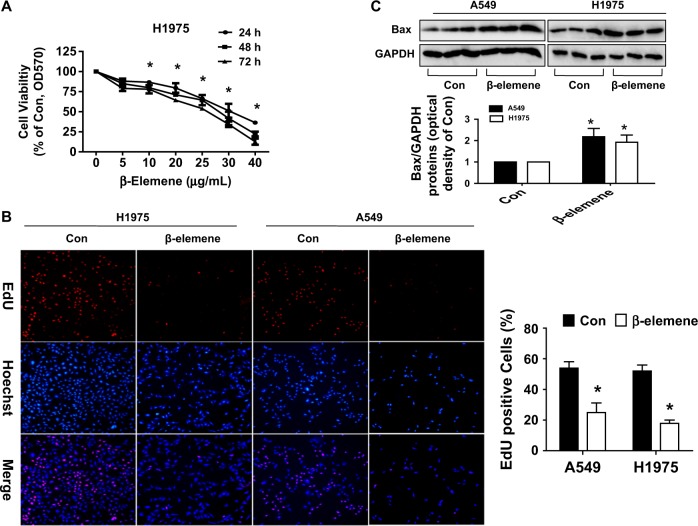
β-Elemene inhibited growth in lung cancer cells. **a** H1975 cells were stimulated with different concentrations of β-elemene for up to 72 h. The cells were collected and processed for MTT assay, as described in the Materials and Methods section. **b** A549 and H1975 cells were treated with β-elemene (25 μg/mL) for 48 h followed by determination of cell growth with the Cell-Light EdU DNA cell proliferation kit. The image was magnified ×10. Hoechst was used to stain all nuclei. At least 3 captured fields were randomly selected, and the percentage of EdU-positive cells = (EdU-positive cells/Hoechst stain cells) × 100. **c** A549 and H1975 cells were treated with β-elemene (30 μg/mL) for 24 h, and the protein levels of Bax were measured by Western blot analysis. GAPDH was used as loading control. Values and bar graphs are presented as the mean ± SEM or the mean ± SD of Bax/GAPDH of 3 independent experiments. *indicates significant difference from the control group (*P* < 0.05). **indicates significant difference between combination treatment and treatment with β-elemene alone (*P* < 0.05)

We previously showed that β-elemene inhibited the growth of human lung cancer cells^7^. In the current study, we further assessed the relative contribution to inhibition attributed to β-elemene. Compared with that of the untreated control cells, the growth of NSCLC H1957 cells treated with β-elemene was significantly inhibited, as determined by MTT assay (Fig. 1a). Cell growth was further examined by Cell-Light EdU DNA cell proliferation assay. EdU, an indicator of DNA synthesis, was used to detect proliferation in A549 and H1975 cells (Fig. 1b). Hoechst was used to stain the nuclei (Fig. 1b). The results showed that the percentages of EdU-positive cells in the β-elemene-treated group were 24.93% ± 6.22 in A549 cells and 17.92% ± 2.11 in H1975 cells, which were reduced compared to those in the control group (53.89% ± 4.22 in A549 cells and 52.02% ± 3.96 in H1975 cells) (Fig. 1b). In line with this, in examining the nature of cell cycle arrest, we previously observed that the β-elemene-treated group led to a decrease in the proportion of cells in G0/G1 phases, as detected by flow cytometry. Concomitantly, the population of the cells at the S phase was significantly induced after treatment with β-elemene in A549 cells^7^. Moreover, β-elemene induced Bax protein expression, suggesting that apoptosis was induced by β-elemene, which was considered a part of cell growth inhibition (Fig. 1c). The aforementioned results further indicated the inhibitory effects of β-elemene on lung cancer growth.

### β-Elemene decreased phosphorylation of Stat3 and increased protein expression of FOXO3a via Stat3

**Fig. 2:**
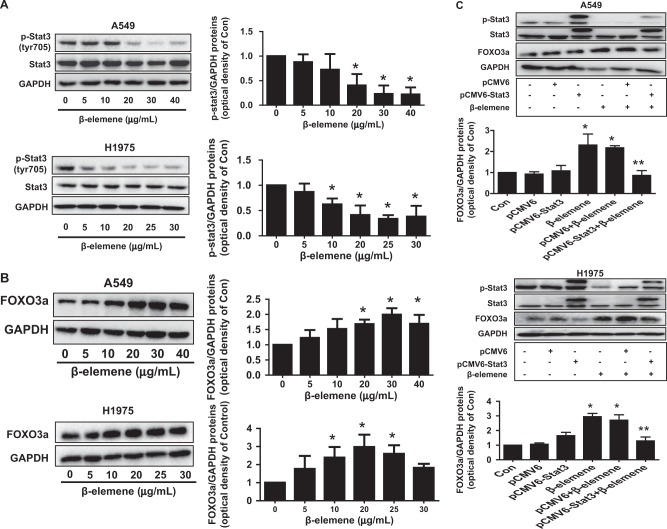
β-Elemene decreased phosphorylation of Stat3 and increased protein expression of FOXO3a via Stat3. **a** A549 and H1975 cells were exposed to the increased concentration of β-elemene for 24 h, and the phosphorylation and protein expression of Stat3 were measured by western blot analysis. GAPDH was used as loading control. The bar graphs represent the mean ± SD of p-Stat3/GAPDH of 3 independent experiments. **b** A549 and H1975 cells were exposed to the increased concentration of β-elemene for 24 h, and the protein expression of FOXO3a was measured by western blot analysis. GAPDH was used as loading control. The bar graphs represent the mean ± SD of FOXO3a/GAPDH of 3 independent experiments. **c** A549 and H1975 cells were transfected with the control or Stat3a expression vectors for 24 h and then exposed to β-elemene (25 μg/mL) for an additional period of 24 h. The phosphorylation and protein expression levels of Stat3 and FOXO3a were subsequently examined by western blot analysis. GAPDH was used as loading control. The bar graphs represent the mean ± SD of FOXO3a/GAPDH of 3 independent experiments. *indicates significant difference compared with the untreated control group (*P* < 0.05); **indicates significant difference between combination treatment and treatment with β-elemene alone (*P* < 0.05)

To gain insight into the potential signaling pathways involved in β-elemene-inhibited growth of lung cancer cells, we evaluated the effect of β-elemene on Stat3 signaling. Stat3 is recognized as a transcription factor that modulates the transcription of various genes to regulate important biological functions, including cell proliferation, differentiation, survival, angiogenesis, immune response, and cancer survival^8,9^. We observed that β-elemene decreased the phosphorylation of Stat3 but exerted no effect on total Stat3 protein expression in A549 and H1975 cells in a dose-dependent fashion within 24 h (Fig. 2a). By contrast, β-elemene increased the protein expression of FOXO3a, an important transcriptional regulator and tumor suppressor^17^ dose-dependently with substantial induction observed at 10–20 μg/mL in H1975 and A549 cells (Fig. 2b). Notably, exogenously expressed Stat3 was shown to overcome the β-elemene-increased protein expression of FOXO3a (Fig. 2c). The excessive expression of the Stat3 gene also induced phosphorylation of Stat3 (Fig. 2c). This finding indicated the possible direct effect of inactivation of Stat3 by β-elemene, resulting in the induction of FOXO3a, a downstream molecule of Stat3, in this process.

### β-Elemene affected the interaction between miRNA155-5p and FOXO3

**Fig. 3:**
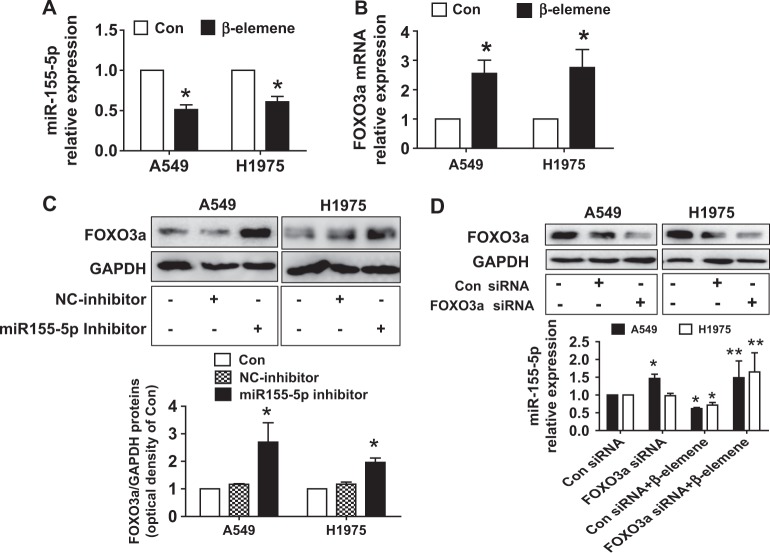
β-Elemene affects the interaction between miRNA155-5p and FOXO3. **a**–**b** A549 and H1975 cells were treated with β-elemene (25 μg/mL) for 24 h, and the mRNA levels of miRNA155-5p and FOXO3a were measured by qRT-PCR. **c** A549 and H1975 cells were treated with the control or the miRNA155-5p inhibitor (50 nM) for 24 h, and the FOXO3a protein levels were measured by western blot analysis. GAPDH was used as loading control. The bar graphs represent the mean ± SD of FOXO3a/GAPDH of 3 independent experiments. **d** A549 and H1975 cells were transfected with the control or FOXO3a siRNA for 24 h. The protein levels of FOXO3a were then measured by western blot analysis, and the mRNA levels of miR155-5p were measured by qRT-PCR. The inserts in the upper panels are FOXO3a protein blots. *indicates significant difference compared with the untreated control group (*P* < 0.05); **indicates significant difference between combination treatment and treatment with β-elemene alone (*P* < 0.05)

**Fig. 4:**
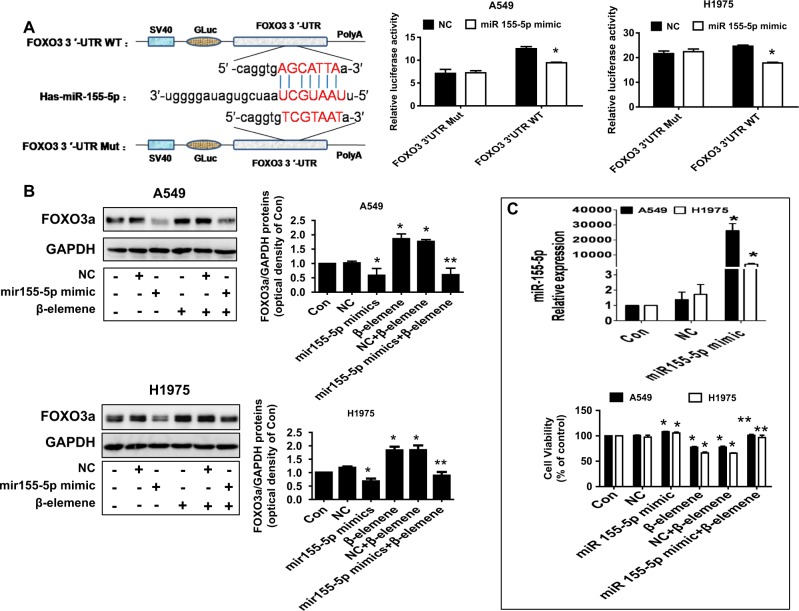
miR155-5p mimics reduced luciferase activity, resisting β-elemene-increased FOXO3a protein levels and cell growth inhibition. **a** A549 and H1975 cells were transfected with mutant and wild-type FOXO3a vectors containing the putative miR155-5p recognition site in the 3′UTR region and miR155-5p mimics for 24 h. Luciferase activity was then measured using the Secrete-Pair™ Dual Luminescence Assay Kit as described in the Materials and Methods. The upper panels are predicated target sites for miR155-5p in the FOXO3a mRNA 3′UTR and FOXO3a mut sequences. **b**–**c** A549 and H1975 cells were treated with the control and miR155-5p mimics (50 nM) for 24 h before exposure of the cells to β-elemene for an additional period of 24 h. FOXO3a protein levels and cell growth were subsequently examined by western blot analysis and MTT assays, respectively. GAPDH was used as loading control. The bar graphs represent the mean ± SD of FOXO3a/GAPDH of 3 independent experiments. *indicates significant difference compared with the untreated control group (*P* < 0.05); **indicates significant difference between combination treatment and treatment with β-elemene alone (*P* < 0.05)

To further dissect the mechanism, we determined the expression and potential interaction of downstream effectors of Stat3 regulated by β-elemene. We evaluated this potential interaction because of the crucial role of miRNAs and that of the transcription factor FOXO3a as either oncogenes or tumor suppressor genes in human cancer as well as links to Stat3^23,43–45^. We showed that β-elemene inhibited miRNA155-5p while increasing FOXO3a mRNA levels in A549 and H1975 cells (Fig. 3a, b). We also found that the miRNA155-5p inhibitor increased FOXO3a protein levels (Fig. 3c). Conversely, we observed that silencing FOXO3a using siRNA methods reduced miR155-5p mRNA levels, suggesting a reciprocal interaction between FOXO3a and miR155-5p in this process (Fig. 3d). In addition, to further assess the link of FOXO3a and miR155-5p, we conducted dual luciferase activity assays using the FOXO3a cDNA sequence containing the putative miR155-5p recognition site in the 3′UTR. Our results demonstrated that miR155-5p mimics reduced the luciferase activity of the FOXO3a wild-type fragment but not that of the mutated one (Fig. 4a). This finding implies potential direct binding in the FOXO3a 3-UTR region and reduction of FOXO3a expression by miR155-5p. Moreover, miR155-5p mimics resisted the β-elemene-increased FOXO3a protein levels (Fig. 4b). We found that miR155-5p mimics antagonized the β-elemene-inhibited cell growth (Fig. 4c). Together, the aforementioned results indicated the following: reciprocal interaction was present between FOXO3a and miR155-5p; the 3′-untranslated region of FOXO3a mRNA was targeted by miR155-5p, and miR155-5p was reduced while FOXO3a was induced. All of these factors contributed to the overall effects of β-elemene in this process.

### β-Elemene induced IGFBP1 protein, mRNA expression, and promoter activity via induction of FOXO3a

**Fig. 5:**
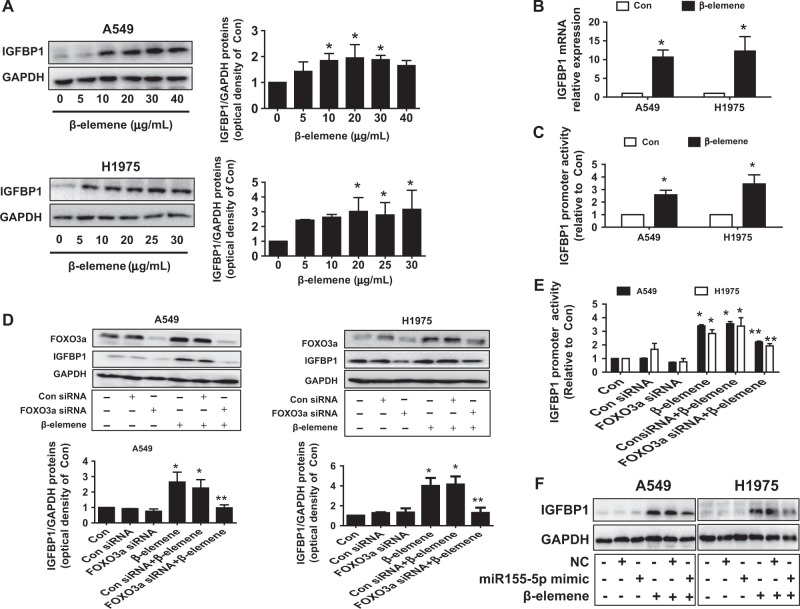
β-elemene induced IGFBP-1 mRNA and protein expression levels and promoter activity via induction of FOXO3a. **a**–**b** A549 and H975 cells were exposed to the increased concentration of β-elemene or β-elemene (25 μg/mL) for 24 h, and the protein and mRNA expression levels of IGFBP1 were measured by western blot analysis and qRT-PCR, respectively. **c** A549 and H1975 cells were transfected with the wild-type human IGFBP1 promoter reporter construct ligated to the luciferase reporter gene and internal control-secreted alkaline phosphatase for 24 h followed by treatment with β-elemene (25 μg/mL) for an additional period of 24 h. The promoter activities were then determined using the Secrete-Pair Dual Luminescence Assay Kit as described in the Materials and Methods. **d**–**e** A549 and H1975 cells were transfected with the control or FOXO3a siRNA, the wild-type human IGFBP1 promoter reporter construct ligated to the luciferase reporter gene, and internal control-secreted alkaline phosphatase for 24 h before the cells were exposed to β-elemene (25 μg/mL) for an additional period of 24 h. IGFBP1 protein expression and promoter activity were subsequently examined by western blot analysis and the Secrete-Pair Dual Luminescence Assay Kit as described in the Materials and Methods. GAPDH was used as loading control. **f** A549 and H1975 cells were treated with the control and miR155-5p mimics (50 nM) for 24 h before the cells were exposed to β-elemene (25 μg/mL) for an additional period of 24 h. IGFBP1 protein levels were then examined by western blot analysis. GAPDH was used as loading control. The bar graphs represent the mean ± SD of IGFBP1/GAPDH of 3 independent experiments. *indicates significant difference compared with the untreated control group (*P* < 0.05); **indicates significant difference between combination treatment and treatment with β-elemene alone (*P* < 0.05)

**Fig. 6:**
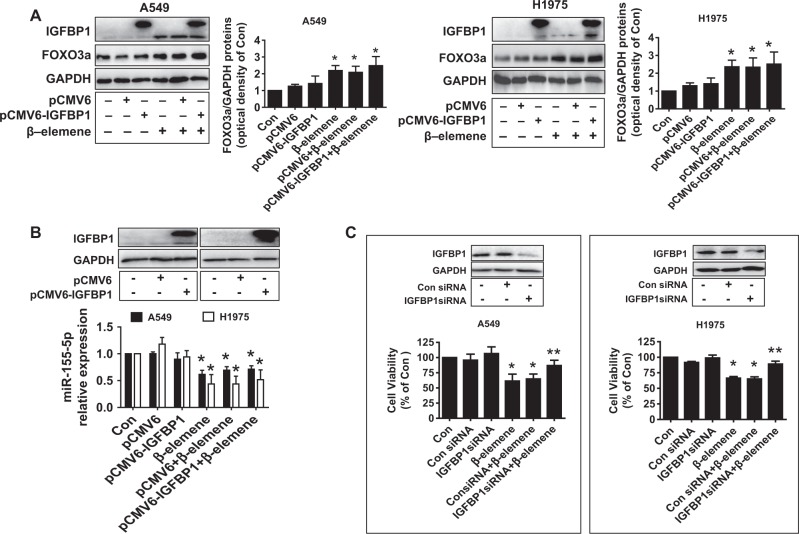
Exogenously expressed IGFBP1 exerted no effect on miR155-5p and FOXO3a expression levels but overcame the effect of β-elemene on cell growth inhibition. **a**–**c** A549 and H1975 cells were transfected with the control or IGFBP1 expression vector or IGFBP1 siRNA for 24 h before exposure of the cells to β-elemene (25 μg/mL) for an additional period of 24 h. Subsequently, miR155-5p expression, FOXO3a expression, and cell growth were determined by qRT-PCR, western blot analysis, and MTT assays, respectively. The inserts in the upper panels are protein expression levels of IGFBP1. GAPDH was used as an internal control. *indicates significant difference compared with the untreated control group (*P* < 0.05); **indicates significant difference between combination treatment and treatment with β-elemene alone (*P* < 0.05)

To further identify relevant targets and gain insight into the biological significance of the interaction between FOXO3a and miR155-5p, we determined the role of IGFBP1, which is secreted by the liver and exerts a protective role in the development of cancer^28–30^. We demonstrated that β-elemene increased not only the protein and mRNA levels of IGFBP1, as determined by western blot analysis and qRT-PCR (Fig. 5a, b), respectively, but the IGFBP1 gene promoter activity as well, as determined by Secrete-Pair™ Dual Luminescence Assay Kit (Fig. 5c). We then investigated the role of FOXO3a to examine the functional relevance of the transcriptional factors involved in the upregulation of IGFBP1 expression. Reports showed that the IGFBP1 promoter contained FOXO binding sites and that FOXOs could regulate IGFBP1 gene expression and its downstream signaling^46,47^. We observed that silencing of FOXO3a neutralized the effect of β-elemene on not only IGFBP1 protein expression but also promoter activity in both A549 and H1975 cells (Fig. 5d, e). This implied the strong interaction and potential recruitment of FOXO3a on the transcriptional regulation of IGFBP1. In accordance with this observation, miR155-5p mimics also blocked the effect of β-elemene on IGFBP1 expression (Fig. 5f). We further evaluated the potential feedback regulatory loops of IGFBP1 and found that excessively expressed IGFBP1 exerted no significant effect on the expression levels of miR155-5p and FOXO3a treated with β-elemene (Fig. 6a, b). Together, the aforementioned results indicated that miR155-5p and FOXO3a, acting as upstream signals of IGFBP1, regulated IGFBP1 expression in NSCLC cells. We further characterized the ability of IGFBP1 to regulate cell growth. We observed that exogenously expressed IGFBP1 overcame the effect of β-elemene on cell growth inhibition (Fig. 6c), suggesting the critical role of IGFBP1 induction in this process.

### Antitumor efficacy of β-elemene in a nude mouse model

**Fig. 7:**
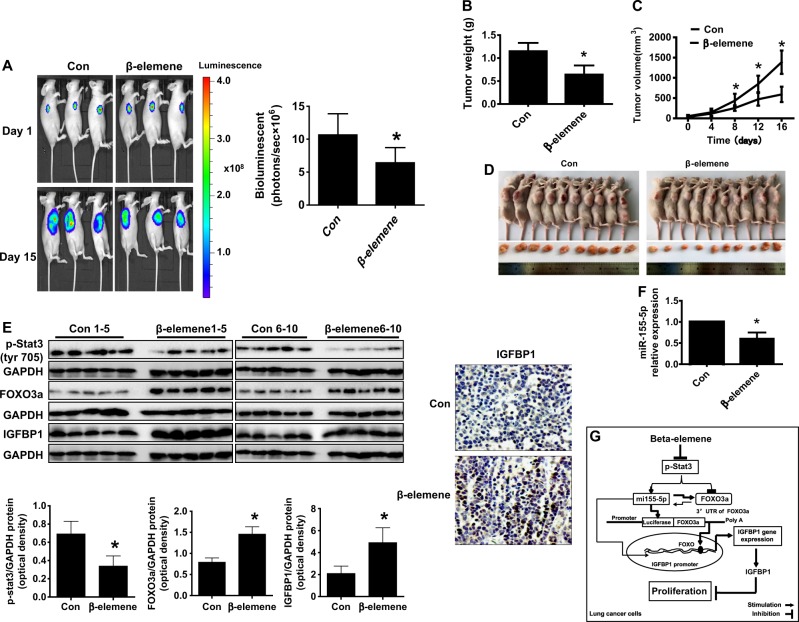
Antitumor efficacy of β-elemene in a nude mouse model. Mice (*n* = 10/group) were divided into 2 groups (the control group (saline) and the β-elemene-treated group (75 mg/kg)) and given treatment 7 days after tumor cells were introduced via intraperitoneal injection for up to 16 days. **a** Xenografts were assessed by in vivo bioluminescence imaging on the first and last days of the experiments (days 1 and 16, respectively). Tumor growth was monitored by injecting luciferin into the mice followed by measurement of bioluminescence with the IVIS Imaging System. Imaging and quantification of signals were controlled using the Living Image acquisition and analysis software, as described in the Materials and Methods. Representative images are shown. **b**, **c** Xenografts were harvested on day 16, and the volumes and weights of the tumors were measured. **d** Photographs of the vehicle- and β-elemene-treated xenografts derived from nude mice are shown. **e**–**f** At the end of the experiments, xenografted tumors were isolated from individual animals; the corresponding lysates were processed, and p-Stat3, FOXO3a, and IGFBP1 protein levels were detected by western blot analysis with the indicated antibodies, immunohistochemistry for IGFBP1 staining, and levels of miR155-5p by qRT-PCR, as described in the Materials and Methods. Scale bar: 50 μM. The bar graphs represent the weights and volumes of the tumors in mice as the mean ± SD. *indicates significant difference from the untreated control (*p* < 0.05). **g** Diagram showing that β-elemene increases IGFBP1 gene expression via inactivation of Stat3 followed by reciprocal regulation and interaction between FOXO3a and miRNA155-5p. This effect leads to the inhibition of human lung cancer cell growth.

Given that the in vitro data showed the effect of β-elemene on human lung cancer cells, we further evaluated the effect of β-elemene on a xenograft tumor model. Luciferase-expressing A549 cells (2 × 10^6^) were injected intraperitoneally into nude mice followed by intraperitoneal injection of D-luciferin. Mice bearing xenografted tumors were treated with control or β-elemene via intraperitoneal injection for up to 16 d. Compared with the control group, the β-elemene-treated group demonstrated a significant inhibitory effect on growth, as assessed by the Xenogen IVIS200 System (Fig. 7a). In addition, we found that the tumor weight and size of the β-elemene-treated group were significantly reduced relative to those of the control group (Fig. 7b–d). Consistent with the results in vitro, FOXO3a and IGFBP1 protein expression levels were induced in the β-elemene-treated group relative to those in the control group; meanwhile, Stat3 phosphorylation and miR155-5p mRNA levels from fresh tumors harvested from the aforementioned experiments were reduced in the β-elemene-treated group relative to those in the control group (Fig. 7e, f). Notably, immunohistochemistry showed increased IGFBP1 expression relative to that of the control (Fig. 7e).

## Discussion

In this study, we presented further evidence regarding protein regulation, miRNA expression, cell proliferation, and xenograft experiments in vivo to characterize the critical role of IGFBP1 as a tumor suppressor in mediating the anti-lung cancer effects of β-elemene. Our results indicate that inactivation of Stat3, followed by the interaction and regulation of miR155-5p and FOXO3a, contributed to the induction of IGFBP1 expression by β-elemene both in vitro and in vivo.

Numerous studies have been conducted to explore the mechanisms underlying the anti-cancer effects of using natural plants. Extracted from *Rhizoma zedoariae*, β-elemene has been widely used for its antitumor activity against a broad range of cancer types^3,4,48^. The studies we conducted, along with other studies, suggested the involvement of the antitumor effects of β-elemene in the regulation of multiple signaling pathways and targets^6,7,49^; regardless, the true molecular mechanisms underlying the anti-lung cancer effect have yet to be determined. Our current results confirm the anti-lung cancer effects of β-elemene and indicate that this agent can be used as a potential therapeutic modality in lung cancer.

We demonstrated the role of transcription factor Stat3, suggesting that inactivation of Stat3 was involved in the β-elemene inhibition of lung cancer growth. As an important signaling molecule for many cytokines and growth factor receptors, constitutive activation of Stat3 occurs at a high frequency in many types of cancer. Our findings suggest that inactivation of Stat3 is involved in mediating the regulation of two important downstream molecules, the transcription factor FOXO3 and miR155-5p, thereby increasing IGFBP1 expression and inhibiting cell growth. This finding also implies that direct inactivation of Stat3 by β-elemene was required, resulting in the induction of FOXO3a and reduction of miR155-5p. In fact, Stat3 can be activated by a wide range of ligands via binding to cytokine, growth factor, or G-protein-coupled receptors either directly or by favoring the action of upstream regulators, leading to increased cell proliferation, differentiation, apoptosis, angiogenesis, and immune response^50^. Thus, the underlying mechanisms, such as whether β-elemene inhibits the upstream molecules of Stat3 resulting affected Stat3-associated signaling, remain inconclusive, and more experiments are still needed to elucidate them. In accordance with this, inactivation and inhibition of Stat3 signaling have been shown to be involved in the inhibition of cell growth and progression in several cancer types, including lung cancer^10,51–53^. This finding suggested that targeting Stat3 could be used in lung cancer treatment.

As a bona fide pleiotropic tumor suppressor and factor functioning downstream of the essential tumor kinase signaling cascades, FOXO3 was shown to be involved in various biological functions, including cell proliferation, differentiation, cell survival, senescence, DNA damage repair, and cell cycle control via the regulation of relevant gene expression^16,17,45,54^. The function roles of miR155-5p have thus far been reported to be involved in cancer growth and progression in different cancer types^25–27^. Our results indicate that a reciprocal interaction is present between FOXO3a and miR155-5p, which leads to the β-elemene-reduced miR155-5p and -induced FOXO3a. The induction of FOXO3a and inhibition of miR155-5p participated in the regulation of other genes and signals that modulated cancer cell proliferation, which demonstrated the crucial roles of these molecules in other studies^25,45^. Thus, FOXO3a and miR155-5p could be used as potential targets in certain cancer treatment modalities. Further studies on the mechanisms by which interaction of FOXO3a and miR155-5p interfere with lung cancer oncogenesis need to be conducted.

We also demonstrated the important role of FOXO3a and miR155-5p in mediating the effect of β-elemene on IGFBP1 expression, suggesting a strong interaction and the transcriptional or/and translational regulation of IGFBP1 by FOXO3a and miR155-5p. Reports linking FOXO and miRNAs to IGFBP1 are limited^36,45,55^. Meanwhile, our current results reveal a regulatory axis linking FOXO3a and miR155-5p to IGFBP1, all of which have been involved in the control of biological functions, including lung cancer growth. The physical binding between miR155-5p and FOXO3a, as well as the 3′-untranslated region of FOXO3a mRNA, was targeted by miR155-5p, suggesting that miR155-5p could regulate FOXO3a expression. Interestingly, silencing of FOXO3a also resisted β-elemene-inhibited miR155-5p mRNA levels, the reasons for which remain unclear. Regardless, FOXO3a and miR155-5p exhibited a reciprocal interaction in the current study, thus influencing IGFBP1 expression. Although the direct binding of FOXO factors on the IGFBP1 promoter region had been reported previously^46^, we reasoned that more experiments are still required to determine if there is recruitment of FOXO3a into the IGFBP1 promoter and whether there is physical binding between FOXO3a and IGFBP1; miR155-5p can bind to the IGFBP1 3′-UTR, thereby inhibiting IGFBP1 expression.

We observed that increased IGFBP1 expression was involved in β-elemene-inhibited cell proliferation in this process. In addition to preventing IGF from binding to IGF-I receptors, IGF-independent actions such as transcriptional regulation, binding to non-IGF molecules, and regulation of other gene expression levels have been reported, and these processes were shown to be involved in oncogenesis, growth, progression, and metastasis^30,32–34^. These findings, together with our results, highlight the tumor suppressor role of IGFBP1 in lung cancer. However, contradictory findings have also been observed in other cancer types, such as prostate cancer^38,39^ and endometrial cancer^40^. Thus, the true function of IGFBP1 acting as a tumor suppressor or oncogene may depend on the capacity of the context and environment exposed, as well as the cancer type examined, which require further elucidation. Nevertheless, our study suggests that IGFBP1 can be regarded as a potential biomarker for lung cancer survival and that increased IGFBP1 could enhance compatibility with current treatment regimens for lung cancer.

Our in vivo data were consistent with the findings of the in vitro study, confirming the antitumor effects of β-elemene in lung cancer and regulation of miR155-5p, FOXO3a, and IGFBP1 expression. The doses of β-elemene used were based on our previous reports and other studies^7,56–58^, demonstrating substantial inhibition of tumor growth in vivo. Despite these findings, more experimental approaches are needed to clarify the true role of IGFBP1 in lung cancer cell biology by using stable transfection of cells with shRNA of the IGFBP1 gene in animal models.

In conclusion, our results show that β-elemene increases IGFBP1 gene expression via inactivation of Stat3 followed by reciprocal regulation and interaction between miRNA155-5p and FOXO3a. This effect ultimately leads to the inhibition of human lung cancer cell growth (Fig. 7g). Our data provide a new molecular mechanistic connection between FOXO3a and miRNA155-5p, as well as the subsequently induced IGFBP1-mediated mechanism underlying the anti-lung cancer effects of β-elemene.

## References

[1] Siegel RL, Miller KD, Jemal A (2017). Cancer Statistics, 2017. Ca. Cancer J. Clin..

[2] Chang GC (2005). Activity of gefitinib in advanced non-small-cell lung cancer with very poor performance status. Invest. New Drugs.

[3] Zhu T (2015). Reversion of malignant phenotypes of human glioblastoma cells by beta-elemene through beta-catenin-mediated regulation of stemness-, differentiation- and epithelial-to-mesenchymal transition-related molecules. J. Transl. Med..

[4] Liu M (2017). beta-Elemene attenuates atherosclerosis in apolipoprotein E-deficient mice via restoring NO levels and alleviating oxidative stress. Biomed. Pharmacother..

[5] Wang Z, Li Y, Zhou F, Piao Z, Hao J (2018). beta-elemene enhances anticancer bone neoplasms efficacy of paclitaxel through regulation of GPR124 in bone neoplasms cells. Oncol. Lett..

[6] Liu Y (2017). beta-elemene regulates endoplasmic reticulum stress to induce the apoptosis of NSCLC cells through PERK/IRE1alpha/ATF6 pathway. Biomed. Pharmacother..

[7] Zhao S (2015). beta-elemene inhibited expression of DNA methyltransferase 1 through activation of ERK1/2 and AMPK alpha signalling pathways in human lung cancer cells: the role of Sp1. J. Cell. Mol. Med..

[8] Yang XW (2017). STAT3 overexpression promotes metastasis in intrahepatic cholangiocarcinoma and correlates negatively with surgical outcome. Oncotarget.

[9] Xu YZ (2017). The long noncoding RNA FOXCUT promotes proliferation and migration by targeting FOXC1 in nasopharyngeal carcinoma. Tumour Biol..

[10] Miyata H (2017). Combination of a STAT3 inhibitor and an mTOR inhibitor against a Temozolomide-resistant Glioblastoma cell line. Cancer Genom. Proteom..

[11] Saengboonmee C (2017). Metformin exerts antiproliferative and anti-metastatic effects against Cholangiocarcinoma cells by targeting STAT3 and NF-kB. Anticancer Res..

[12] Jia L (2016). Dihydroartemisinin as a putative STAT3 inhibitor, suppresses the growth of head and neck squamous cell carcinoma by targeting Jak2/STAT3 signaling. PLoS ONE.

[13] Wang Y, Shen Y, Wang S, Shen Q, Zhou X (2017). The role of STAT3 in leading the crosstalk between human cancers and the immune system. Cancer Lett..

[14] Huang H, Tindall DJ (2007). Dynamic FoxO transcription factors. J. Cell. Sci..

[15] Carbajo-Pescador S, Mauriz JL, Garcia-Palomo A, Gonzalez-Gallego J (2014). FoxO proteins: regulation and molecular targets in liver cancer. Curr. Med. Chem..

[16] Bigarella CL (2017). FOXO3 transcription factor is essential for protecting hematopoietic stem and progenitor cells from oxidative DNA damage. J. Biol. Chem..

[17] Wang X, Hu S, Liu L (2017). Phosphorylation and acetylation modifications of FOXO3a: Independently or synergistically?. Oncol. Lett..

[18] Liu Y (2018). Critical role of FOXO3a in carcinogenesis. Mol. Cancer.

[19] Zheng F (2015). Baicalein increases the expression and reciprocal interplay of RUNX3 and FOXO3a through crosstalk of AMPK alpha and MEK/ERK1/2 signaling pathways in human non-small cell lung cancer cells. J. Exp. Clin. Cancer Res..

[20] Ni D (2014). Downregulation of FOXO3a promotes tumor metastasis and is associated with metastasis-free survival of patients with clear cell renal cell carcinoma. Clin. Cancer Res..

[21] Kops GJ (2002). Forkhead transcription factor FOXO3a protects quiescent cells from oxidative stress. Nature.

[22] Li Z, Zhang H, Chen Y, Fan L, Fang J (2012). Forkhead transcription factor FOXO3a protein activates nuclear factor kappaB through B-cell lymphoma/leukemia 10 (BCL10) protein and promotes tumor cell survival in serum deprivation. J. Biol. Chem..

[23] Bertoli G, Cava C, Castiglioni I (2016). The potential of miRNAs for diagnosis, treatment and monitoring of breast cancer. Scand. J. Clin. Lab. Invest. Suppl..

[24] Yang Y (2017). The clinical use of circulating microRNAs as non-invasive diagnostic biomarkers for lung cancers. Oncotarget.

[25] Chen G (2017). miR-155-5p modulates malignant behaviors of hepatocellular carcinoma by directly targeting CTHRC1 and indirectly regulating GSK-3beta-involved Wnt/beta-catenin signaling. Cancer Cell. Int..

[26] Al-Haidari AA, Syk I, Thorlacius H (2017). MiR-155-5p positively regulates CCL17-induced colon cancer cell migration by targeting RhoA. Oncotarget.

[27] Fu X (2017). MicroRNA-155-5p promotes hepatocellular carcinoma progression by suppressing PTEN through the PI3K/Akt pathway. Cancer Sci..

[28] Major JM, Laughlin GA, Kritz-Silverstein D, Wingard DL, Barrett-Connor E (2010). Insulin-like growth factor-I and cancer mortality in older men. J. Clin. Endocrinol. Metab..

[29] Katanasaka Y (2014). Synergistic anti-tumor effects of a novel phosphatidyl inositol-3 kinase/mammalian target of rapamycin dual inhibitor BGT226 and gefitinib in non-small cell lung cancer cell lines. Cancer Lett..

[30] Kashyap MK (2015). Role of insulin-like growth factor-binding proteins in the pathophysiology and tumorigenesis of gastroesophageal cancers. Tumour Biol..

[31] Kim JJ (2003). Regulation of insulin-like growth factor binding protein-1 promoter activity by FKHR and HOXA10 in primate endometrial cells. Biol. Reprod..

[32] Baxter RC (2014). IGF binding proteins in cancer: mechanistic and clinical insights. Nat. Rev. Cancer.

[33] Ammoun S (2012). Insulin-like growth factor-binding protein-1 (IGFBP-1) regulates human schwannoma proliferation, adhesion and survival. Oncogene.

[34] Kim JC (2016). Complex behavior of ALDH1A1 and IGFBP1 in liver metastasis from a colorectal cancer. PLoS ONE.

[35] Park JH, Rasch MG, Qiu J, Lund IK, Egeblad M (2015). Presence of insulin-like growth factor binding proteins correlates with tumor-promoting effects of matrix metalloproteinase 9 in breast cancer. Neoplasia.

[36] Yang LJ (2016). Inter-regulation of IGFBP1 and FOXO3a unveils novel mechanism in ursolic acid-inhibited growth of hepatocellular carcinoma cells. J. Exp. Clin. Cancer Res..

[37] Tang Q, Wu J, Zheng F, Hann SS, Chen Y (2017). Emodin increases expression of insulin-like growth factor binding protein 1 through activation of MEK/ERK/AMPK alpha and interaction of PPARgamma and Sp1 in lung cancer. Cell. Physiol. Biochem..

[38] Cao Y (2015). Prediagnostic plasma IGFBP-1, IGF-1 and risk of prostate cancer. Int. J. Cancer.

[39] Sharma J (2014). Elevated insulin-like growth factor binding protein-1 (IGFBP-1) in men with metastatic prostate cancer starting androgen deprivation therapy (ADT) is associated with shorter time to castration resistance and overall survival. Prostate.

[40] Shafiee MN (2016). Upregulation of genes involved in the insulin signalling pathway (IGF1, PTEN and IGFBP1) in the endometrium may link polycystic ovarian syndrome and endometrial cancer. Mol. Cell. Endocrinol..

[41] Gong J (2017). Inhibition of FASN suppresses migration, invasion and growth in hepatoma carcinoma cells by deregulating the HIF-1alpha/IGFBP1 pathway. Int. J. Oncol..

[42] Zheng F (2014). p38alpha MAPK-mediated induction and interaction of FOXO3a and p53 contribute to the inhibited-growth and induced-apoptosis of human lung adenocarcinoma cells by berberine. J. Exp. Clin. Cancer Res..

[43] Lin X (2017). PAI-1/PIAS3/Stat3/miR-34a forms a positive feedback loop to promote EMT-mediated metastasis through Stat3 signaling in Non-small cell lung cancer. Biochem. Biophys. Res. Commun..

[44] Oh HM, Yu CR, Dambuza I, Marrero B, Egwuagu CE (2012). STAT3 protein interacts with Class O Forkhead transcription factors in the cytoplasm and regulates nuclear/cytoplasmic localization of FoxO1 and FoxO3a proteins in CD4( + ) T cells. J. Biol. Chem..

[45] Tang Q (2017). Combination of Solamargine and Metformin strengthens IGFBP1 gene expression through inactivation of Stat3 and reciprocal interaction between FOXO3a and SP1. Cell. Physiol. Biochem..

[46] Yalley A, Schill D, Hatta M, Johnson N, Cirillo LA (2016). Loss of interdependent binding by the FoxO1 and FoxA1/A2 forkhead transcription factors culminates in perturbation of active chromatin marks and binding of transcriptional regulators at insulin-sensitive genes. J. Biol. Chem..

[47] Gan L (2005). FoxO-dependent and -independent mechanisms mediate SirT1 effects on IGFBP-1 gene expression. Biochem. Biophys. Res. Commun..

[48] Li J, JunYu, Liu A, Wang Y (2014). beta-Elemene against human lung cancer via upregulation of P53 protein expression to promote the release of exosome. Lung Cancer.

[49] Yu X (2017). beta-elemene inhibits tumor-promoting effect of M2 macrophages in lung cancer. Biochem. Biophy. Res. Commu.

[50] Roca Suarez AA, Van Renne N, Baumert TF, Lupberger J (2018). Viral manipulation of STAT3: evade, exploit, and injure. PLoS Pathog..

[51] Airoldi I (2016). Interleukin-30 promotes breast cancer growth and progression. Cancer Res..

[52] Zhang T (2016). Natural product pectolinarigenin inhibits osteosarcoma growth and metastasis via SHP-1-mediated STAT3 signaling inhibition. Cell Death Dis..

[53] Lee H, Lee HJ, Bae IJ, Kim JJ, Kim SH (2017). Inhibition of STAT3/VEGF/CDK2 axis signaling is critically involved in the antiangiogenic and apoptotic effects of arsenic herbal mixture PROS in non-small lung cancer cells. Oncotarget.

[54] Liang Z (2017). MicroRNA-608 inhibits proliferation of bladder cancer via AKT/FOXO3a signaling pathway. Mol. Cancer.

[55] Tochigi H (2017). Loss of miR-542-3p enhances IGFBP-1 expression in decidualizing human endometrial stromal cells. Sci. Rep..

[56] Wu J (2017). Interplay of DNA methyltransferase 1 and EZH2 through inactivation of Stat3 contributes to beta-elemene-inhibited growth of nasopharyngeal carcinoma cells. Sci. Rep..

[57] Zhou J (2016). Combinatorial antitumor effect of Rapamycin and beta-Elemene in follicular thyroid cancer cells. Biomed. Res. Int..

[58] Li X (2016). beta-elemene sensitizes hepatocellular carcinoma cells to oxaliplatin by preventing oxaliplatin-induced degradation of copper transporter 1. Sci. Rep..

